# The clinical study on treatment of CD19‐directed chimeric antigen receptor‐modified T cells in a case of refractory Richter syndrome

**DOI:** 10.1002/cam4.2193

**Published:** 2019-05-02

**Authors:** Leiming Xia, Yi Wang, Tan Li, Xueying Hu, Qian Chen, Liu Liu, Beilei Jiang, Caixin Li, Hua Wang, Siying Wang, Guanghua Yang, Yangyi Bao

**Affiliations:** ^1^ Department of Hematology The First People`s Hospital of Hefei Hefei China; ^2^ Basic College of Medicine Anhui Medical University Hefei China; ^3^ Shanghai Telebio Biomedical Co. Ltd. Hefei China

**Keywords:** CART: chimeric antigen receptor‐modified T, CART‐19: CD19‐directed chimeric antigen receptor‐modified T cells, cytokines, Richter syndrome

## Abstract

Richter syndrome (RS) indicates the transformation of chronic lymphocytic leukemia (CLL) into an aggressive lymphoma (mostly DLBCL). Richter syndrome is a rare complication with an aggressive clinical course, bearing an unfavorable prognosis. Currently, there is no effective treatment for it. As a novel cellular‐based immune therapy, chimeric antigen receptor‐modified T (CART) cells treatment is gradually used in treating hematological malignancies, especially in CD19^+^ B‐cell malignancy. Therefore, CD19‐directed chimeric antigen receptor‐modified T cells (CART‐19) treatment is promising to be used as a new method for RS patients. In our study, one RS patient expressing high level of CD19 molecule was enrolled in clinical trial; he has received a series of treatments but did not achieve a satisfactory therapeutic effect. The patient totally received 3.55 × 10^8^ autologous CART‐19 cells infusion. After CART‐19 infusion, the mainly clinical side effect was repeated fever. The maximal duration period was 24 days and the highest temperature was 40.1°C. Pancytopenia and significantly serum cytokines level rise were observed, including IFN‐γ, IL‐6, and IL‐10. Before discharge, the level of cytokines reduced to normal levels. In addition, we detected the serum biochemical indices as like K^+^, Ca^2+^, creatinine, and glutamic‐pyruvic transaminase, all of these indices were normal. This showed that there was no tumor necrosis syndrome after treatment. The proportion of B cells in patient's peripheral blood decreased from 72% to 40.2% after infusion, co‐occurring with reduction in lymph nodes and hematopoietic reconstitution. Based on the recent revolution in the therapeutic landscape for hematological malignancies including B‐cell lymphomas, CART‐CD19 cell therapy as a new therapeutic option for RS might be available in the coming years. It aims to reduce patient's tumor burden, prolong their survival time, and provide opportunities for other sequential therapy such as chemotherapy and bone marrow transplantation.

## 1 INTRODUCTION

Richter syndrome (RS) was defined as the development of an aggressive large cell lymphoma in initially diagnosed CLL patients, which contains two sub‐types of the diffuse large B‐cell lymphoma (DLBCL) and the Hodgkin lymphoma (HL) variant.^1^

RS is one of the lymphocytic malignancies with aggressive disease and poor outcome.^2^ Few therapeutic strategies for RS were alternative. The overall survival (OS) is <10 months, only 5%‐20% of RS patients get complete remission (CR) with current chemotherapy treatment.^3^, ^4^, ^5^ Complete remission was achieved by 7%, OS is 21 months for R‐CHOP in RS treatment. The clinical treatment efficacy needs further improvement.^6^

In recent years, some promising agents‐based investigation brings forward the RS treatment to specific target and individualized era.^7^ Specific targeted kinase inhibitors, antagonist of the anti‐apoptotic protein, monoclonal antibody, and blockage of immune checkpoints have been alternative option in clinic. Treatment combining chemotherapy with hematopoietic stem cell transplantation (HSCT) as well as novel‐targeted therapies have shown limited efficacy in RS.

Since most B cell malignancies express CD19, a series of clinic trials based on CD19 targeted therapies were designed. Blinatumomab,^8^ a conjugate of anti‐CD19 and anti‐CD3 monoclonal antibodies, can be used to generate CD19‐targeted bispecific Ab‐armed T cells, which can redirect cytotoxic immunocytes to CD19‐positive tumor cells. Both CR^9^ and relapse/refractory^10^ acute lymphocytic leukemia (ALL) patients are reported could benefit from Blinatumomab with prolonged survival. Actually, Viardot et al. evaluated the effect of 21 evaluable relapse/refractory DLBCL patients received one cycle of blinatumomab, even the ORR and CR was finitely up to 43% and 19%, respectively, but CD19 targeted therapies were still promising future. The ORR was up to 43% and CR was 19%. CART‐19 cells infusion is an alternative promising treatment in B‐cell original tumor, especially in CD19^+^ ALL. Recent publication shown that up to 83% relapsed B‐ALL patients achieved CR status.^11^ However, both of Blinatumomab and CART‐19 therapy meet a huge challenge in lymphoma treatment.

Nevertheless, whether the RS patient could benefit from CART‐19 administration is largely unknown. Here we first describe a patient receiving CART‐19 cells infusion with DLBCL‐type RS who was resistant to multiple chemotherapies.

## 2 PATIENTS AND METHODS

### 2.1 Patient

**Figure 1:**
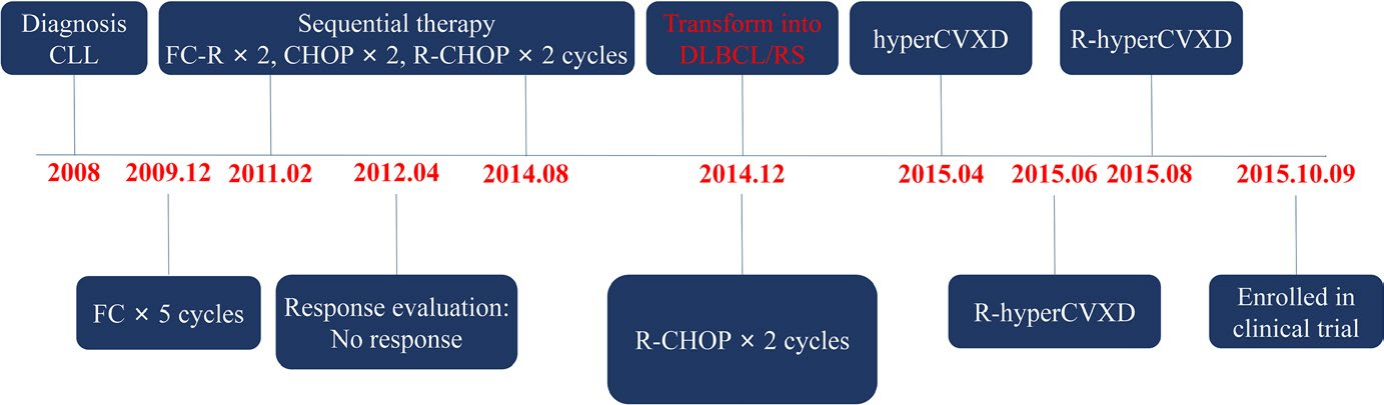
The therapeutic process of the patient suffering Richter Syndrome

**Figure 2:**
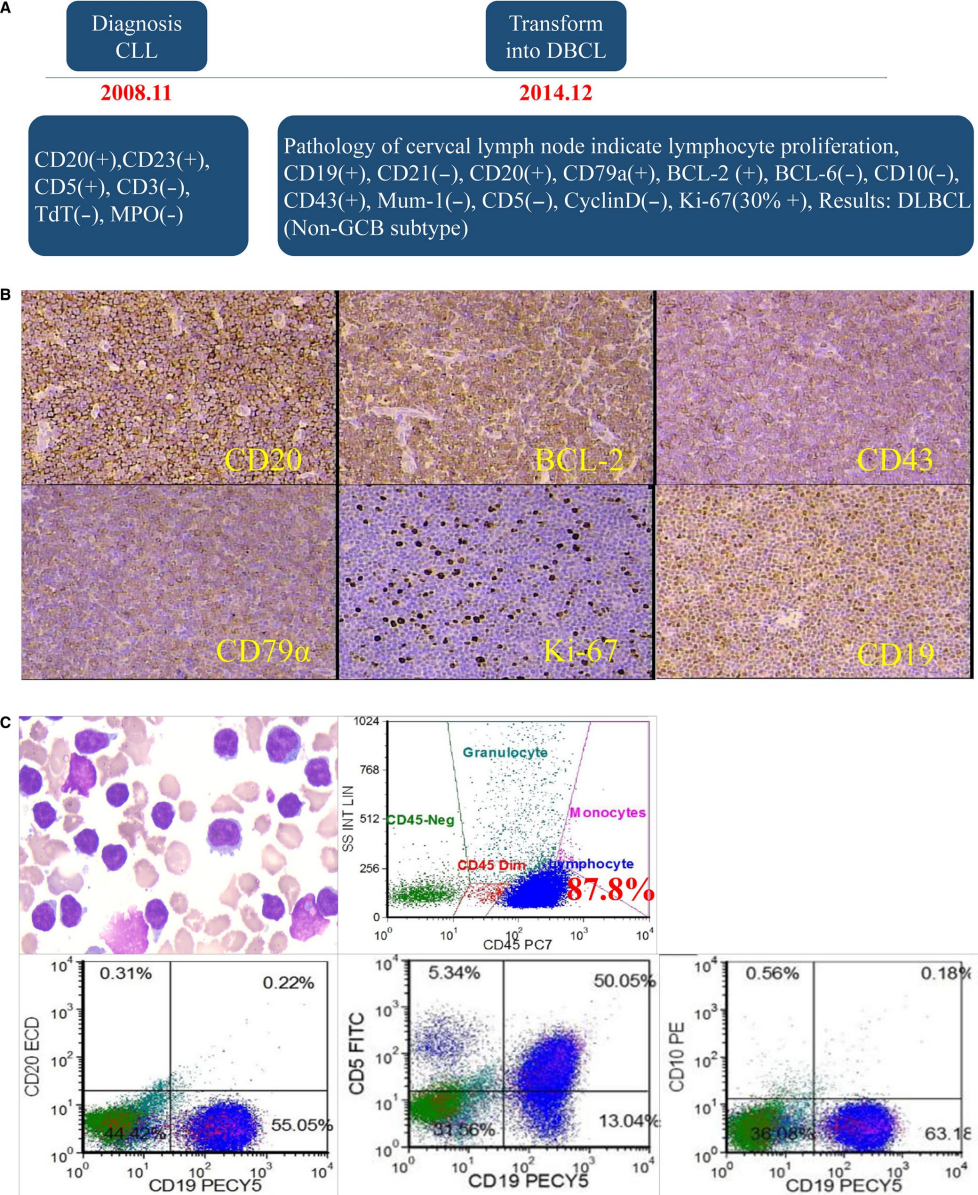
The transformation of the patient from CLL to DLBCL. A, Dynamic results pathology of lymph node during the progression of RS. B, Pathological assay of resected lymph node. C, BM tested by Pathology and immunophenotyping of this RS patient (87.8% cells were CD19 positive; CD5 partial positive, CD10 negative)

A 55‐year‐old male patient presented to local hospital for an enlarged sub‐mandibular lymph nodes with no pain in 2006, and no further examine and treatment were taken. Two years later, the patient referred to the hematology department of our hospital because of progressive systemic lymphadenectasis, splenomegaly, and thrombocytopenia without fever. The count of white blood cells (WBC) and lymphocyte in peripheral blood (PB) was 30 × 10^9^/L and 10 × 10^9^/L, respectively. The phenotype of malignancy of bone marrow (BM) was CD20(+), CD23(+), CD5(+), CD3(−), TdT(−), MPO(−). Finally, the patient was diagnosed as CLL, Rai stage IV, in 2008. Earlier, the patient responded to FC (Fludarabine, CTX) regimen, and then failed to response to the alternate regimen, as shown in Figure 1. In December 2014, the patient suffered from a systemic lymph node enlargement and systemic symptoms (B‐symptoms), including weight loss, fever and night sweats. Immunohistochemical assay revealed the lymphoma cells were CD19(+), CD20(+), CD79a(+), BCL‐2(+), BCL‐6(−), CD10(−), CD43(+), Mum‐1(−), CD5(−), CyclinD(−), Ki‐67(30% +). A disappearance of lymphatic structure and diffuse proliferation of medium‐sized lymphoid cells were found in lymph node. The patient was diagnosed as DLBCL (Non‐germinal center B‐cell type, Non‐GCB) transformed from CLL, also known as RS. Figure 2 reveals the transformation of the RS patient from CLL to DLBCL. After receiving a series of treatments but did not achieved a satisfactory therapeutic effect, the patient was enrolled in our ongoing CART‐19 clinical trial. The details of therapeutic process of the patient suffering RS are summarized in Figure 1.

Following the protocol of CART‐19 clinical trial, the patient received pretreatment of FC (Fludarabine, 30 mg/m^2^, 3 days; CTX, 60 mg/kg, 3 days) without severe side effects, such as inflammation of urinary and digestive tracts, edema. Peripheral neuropathy was also monitored. And then, 3.55 × 10^8^ autologous CART‐19 cells infusion were separated three times, 20% of CART‐19 cells infused on day 0, 30% on day +1, the rest 50% was infused on day +2.

### 2.2 Generation of CAR‐19 lentiviral vectors

CAR‐19 lentiviral vectors were used to generate the effective T lymphocytes. Lentiviral vectors were produced by transfection of HEK293T cells as described [1]. Briefly, HEK293T cells (1 × 10^7^/plate) were cultured in 15‐cm dishes for 24 hours prior to transfection in Dulbecco's modified Eagle's medium (DMEM) with 10% fetal bovine serum (FBS). The culture medium was exchanged 2 hours before transfection (DMEM with 2% FBS). For transient transfection of HEK293T cells, the calcium phosphate precipitation method was used. For each culture dish, a total of 40 μg plasmid DNA was added, including 12 μg envelope plasmid (pMD2.G) encoding VSV‐G, 5 μg packaging plasmid (pMDLg/pRRE), 3 μg of the plasmid producing Rev (pRSV‐Rev) and 20 μg of the transfer vector containing anti‐CD19 scFv,4‐1BB and CD3 zeta fragments. All the lentiviral vectors were collected 48‐72 hours post transfection, concentrated, and stored at −80°C until use.

### 2.3 Preparation, proliferation, transfection, and identification of CART‐19 cells

Autologous CART‐19 cells were generated by T cells derived from the patient. Peripheral blood mononuclear cells (PBMCs) were purified using Ficoll from the PB of patient, Then PBMCs were co‐cultured with supernate of virus for 30 minutes, which contain anti‐CD19 Fcγ, CD28, 41BB fragments. In the following, PBMCs were placed in serum‐free AIM‐V medium and incubated in humidified atmosphere of 5% CO_2_ at 37°C. rh‐IFN‐γ (2000 U/mL) was added on the initial day. After incubation for 24 hours, anti‐CD3 mAb (50 μg/L) and rhIL‐2 (1000 U/mL) were added. The cell suspensions were maintained in subculture with fresh medium with 1000 U/mL rhIL‐2, washed, and refilled every 2‐3 days for 3‐4 weeks. The CART‐19 cells were harvested on day +8.

The transduction efficiency of CAR‐19 plasmid could not be tested in the experiment due to the lack of anti‐anti‐CD19 scFv antibody. Hence, a GFP gene was packed instead of anti‐CD19 scFv fragment in the same plasmid, the parallelly transfected GFP‐positive cells were used to estimate the transduction efficiency of CAR‐19 plasmid.

### 2.4 Collection and arrangement of clinical data

Size of superficial lymph nodes, body temperature, blood pressure, respiration, and pulse of the patient were monitored by three investigators during the hospitalization, respectively, and mean was recorded.

### 2.5 Flow cytometry

The composition of cells in BM was tested by flow cytometry, like CD3^+^ T lymphocytes, CD4^+^ T cells, CD8^+^ T cells, T_reg_ cells, and B cells. Anti‐CD45 (PC7, clone: J33, Beckman Coulter; PE‐cy7, clone: HI30, BD bioscience), anti‐CD3 (ECD, clone: UCHT1, Beckman Coulter), anti‐CD4 (PE, clone: SK3, BD bioscience), CD71 (FITC, clone: M‐A712, BD bioscience), anti‐CD8 (FITC, clone: B9.11, Beckman Coulter), anti‐CD25 (Pacific Blue, clone: B1.49.9, Beckman Coulter), anti‐CD127 (PE‐cy5, clone: A019D5, Biolegend), anti‐CD19(PE‐cy5, clone: J3‐119, Beckman Coulter), anti‐CD20 (ECD, clone: B9E9, Beckman Coulter), anti‐CD5 (FITC, clone: L17F12, BD bioscience), anti‐CD10 (PE clone: ALB1, Beckman Coulter) antibodies were purchased. Flow data were acquired on Beckman Coultor Navios (Beckman Coulter, Atlanta, GA) and analyzed using FCS express version 3 software (De Novo Software, Glendale, CA).

#### 2.5.1 CART‐19 cell mediated cancer lysis assay

**Figure 3:**
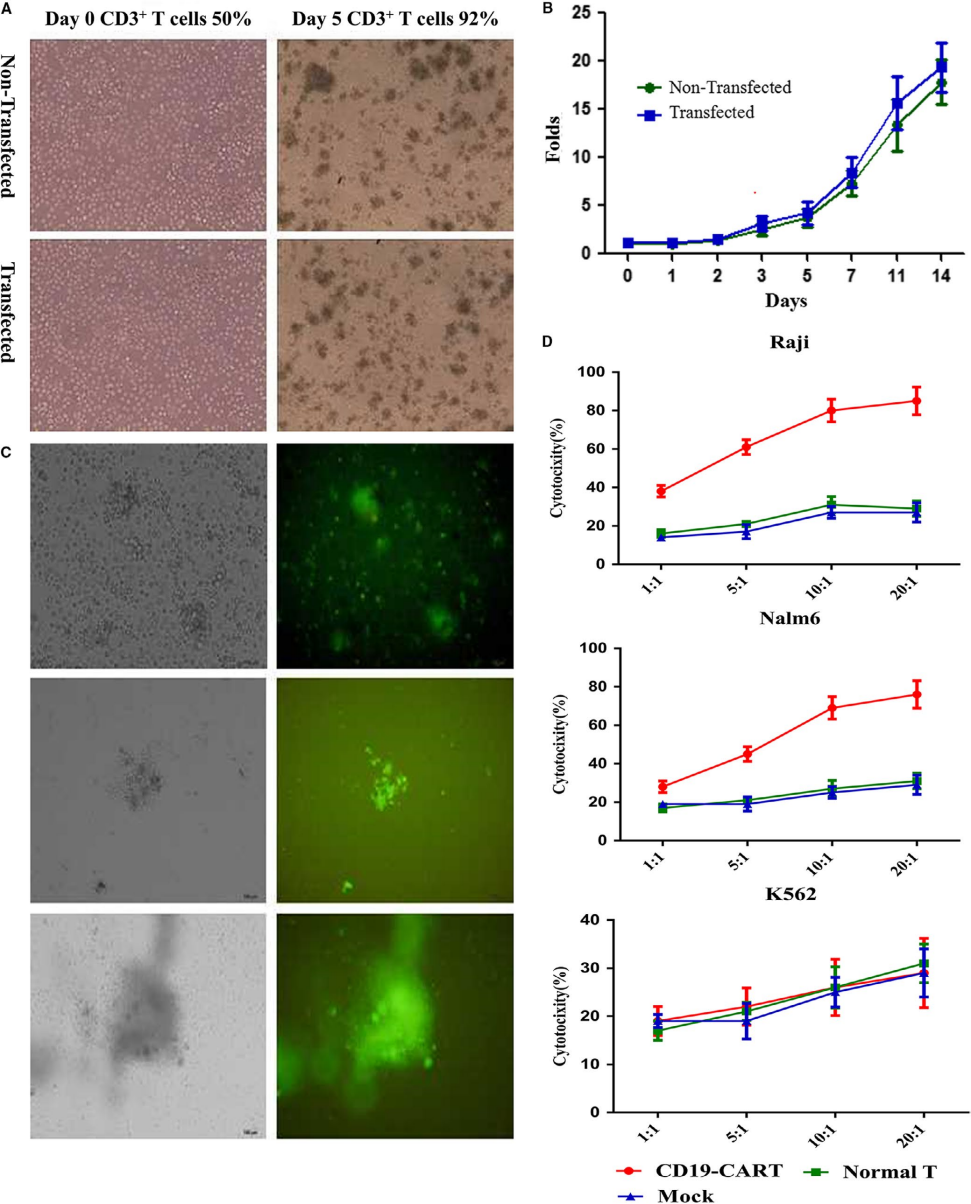
Generation and killing effect of CART‐19 cells in vitro. A‐B, Proliferation of transfected CART‐19 cell in vitro. C, Transfected T cells with control virus, and monitored by green fluorescence of GFP. D, Killing effect of CART‐19 immunocytes in vitro

Cytoplasmic lactate dehydrogenase (LDH) release assay was used to test the cytotoxicity of CART‐19 cells according to the manufacturer's protocol (CytoTox 96 Non‐Radioactive Cytotoxicity Assay, Promega, Madison, WI). Briefly, 5 × 10^4^cells/well target cells(CD19 positive Raji & Nalm‐6 cells and negative K562 cells) were seeded in 96‐well U‐bottom tissue culture plate and exposed to generated CART‐19 immunocytes at effector to target cell ratios indicated in Figure 3D. Twelve hours later, plate were centrifuged and 50 μL supernatant from each well was transferred to a fresh 96‐well plate, 50 μL of the substrate mix was added and incubated in the dark for 30 minutes at room temperature. And then, 50 μL stop solution was added. Maximal release of LDH was performed by incubating the target cells with Lysis Solution. Target cells alone were used as a spontaneous release control. Absorbance was measured at 490 nm using a 96‐well plate reader.

### 2.6 ELISA

Pre‐coated ELISA kits (MultiSciences（Lianke）Biotech, Shanghai, China) were applied to detect the concentration of IL‐2, IL‐6, IL‐8, IL‐10, IL‐12P70, IL‐12/IL‐23P40, TNF‐α, IFN‐γ, and Vascular endothelial growth factor (VEGF) in serum during and after CART‐19 immunocytes infusion, following the manufacturer's instructions. A total of 50 μL of diluent serum, 50 μL of assay buffer and 50 μL biotin‐linked detect antibody was added to 96‐well plates pre‐coated with corresponding primary antibodies. After 2 hours incubation and six times of washing, add 100 μL of diluted Streptavidin‐HRP to each well. And then, the plate was incubated at room temperature for 45 minutes on shaker set at 300 rpm. After incubation and of washing, 100 μL substrate solution was added to each well, incubate for another 30 minutes at room temperature, and stopped. The absorbance at 450 nm was measured using a microplate reader, and recorded. The amount of target in each sample was normalized to the total protein base on the standard curve that linearized by plotting the log of the target concentrations versus the log of the optical density and the best‐fit line can be determined by regression analysis.

### 2.7 PCR

Polymerase chain reaction (qPCR) was used to monitor the DNA fragment of CAR‐19 molecular in patient after the infusion, and reckon the fluctuation of CART‐19 in host, which shown numbers of copies of CAR gene on T cells. The sequences of primers are: (forward primer): 3′‐ATGGCCTTACCAGTGACCGC‐5′, (reverse primer): 3′‐TTAGCGAGGGGGCAGGGCCTGCAT‐5′.

## 3 RESULT

### 3.1 Transfection and killing effect of CART‐19 immunocytes in vitro

PBMCs were proliferated in IL‐2, IFN‐γ enriched system with anti‐CD3 monoclonal antibody. After culturing for 5 days, the percentage of CD3^+^ T lymphocytes in this culture system was up to 92% from 50%. The proliferation of the transfected T cells did not show any difference from the non‐transfected T cells, as shown in Figure 3A,B. Figure 3C shows the transfection efficiency tracked with green fluorescence of GFP. The CART‐19 shows specific killing effect on CD19 positive cells in vitro, like Raji and Nalm‐6 cell lines, but not K562 cells, Figure 3D.

### 3.2 Clinical response

**Figure 4:**
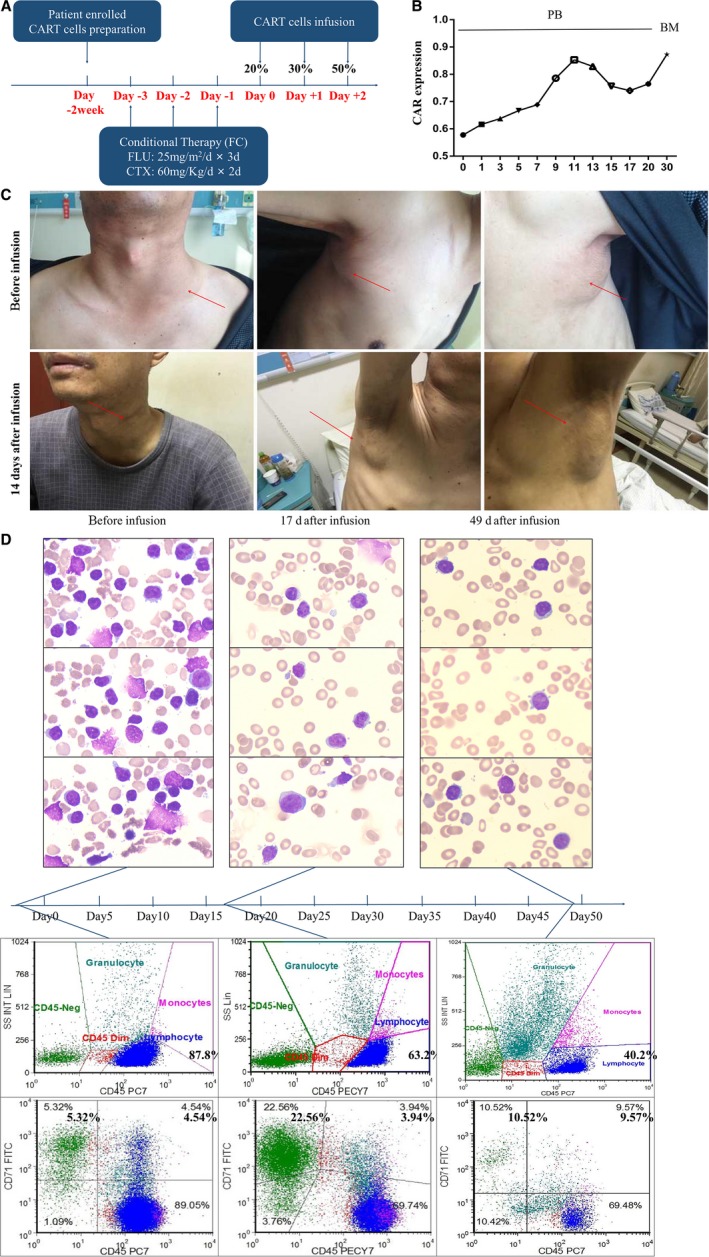
The clinical respond of RS patient receiving CART‐19 immunocytes infusion. A, Protocol of conditional treatment and cells infusion. B, PCR result of circulating CAR‐19 fragment in PBMC (left) and BM(right). C, Photos of superficial lymph node before and after CART infusion. D, Cytological assay and flow cytometry of BM before and after CART infusion

The protocol of immunocytes infusion in this case was designed and shown in Figure 4A. After the pretreatment of FC chemotherapy, 3.55 × 10^8^CART‐19 cells were infused and separated three times as described in methods. Then, the circulating CART‐19 cells in both PBMC and BM were monitored by PCR amplification. The CAR‐19 expression in PBMC could be detected during 20 days after CART‐19 cells infusion, the peak was around day +11 after infusion. More importantly, the expression of CD19‐CAR in BM was maintained in a high level after infusion on day +30, Figure 4B. After 14 days of the immunocytes infusion, the size of superficial was significantly shrunk, but the diameter did not curtail more than 50%, Figure 4C. Also we detected the dynamic variation of malignant cells and hematopoietic reconstitution in BM during the infusion. The cytological assay shows the burden of leukemia cells in BM reduced to 76% on day +17 from 86%, further to 49% on day +49. The results were confirmed by flow, the percentage of lymphoblasts were decreased from 87.8% to 63.2% on day +17, and further decreased to 40.2% on day +49. Moreover, we found percentage of CD71‐positive cells were dramatically increased in CD45^−^ cells from BM after infusion, but the relative is CD71^+^CD45^+^ cells did not show differences to before infusion, no matter on day +17 and +49, and most of the CD71^+^CD45^+^ cells were marked in pink and aquamarine blue, which means they were granulocytes and monocytes, but not lymphocytes, Figure 4D. Correspondingly, the percentage of reticulocytes and account of platelets were recovered and proliferation of WBC was inhibited on day +6, as shown in Figure 6A.

**Figure 5:**
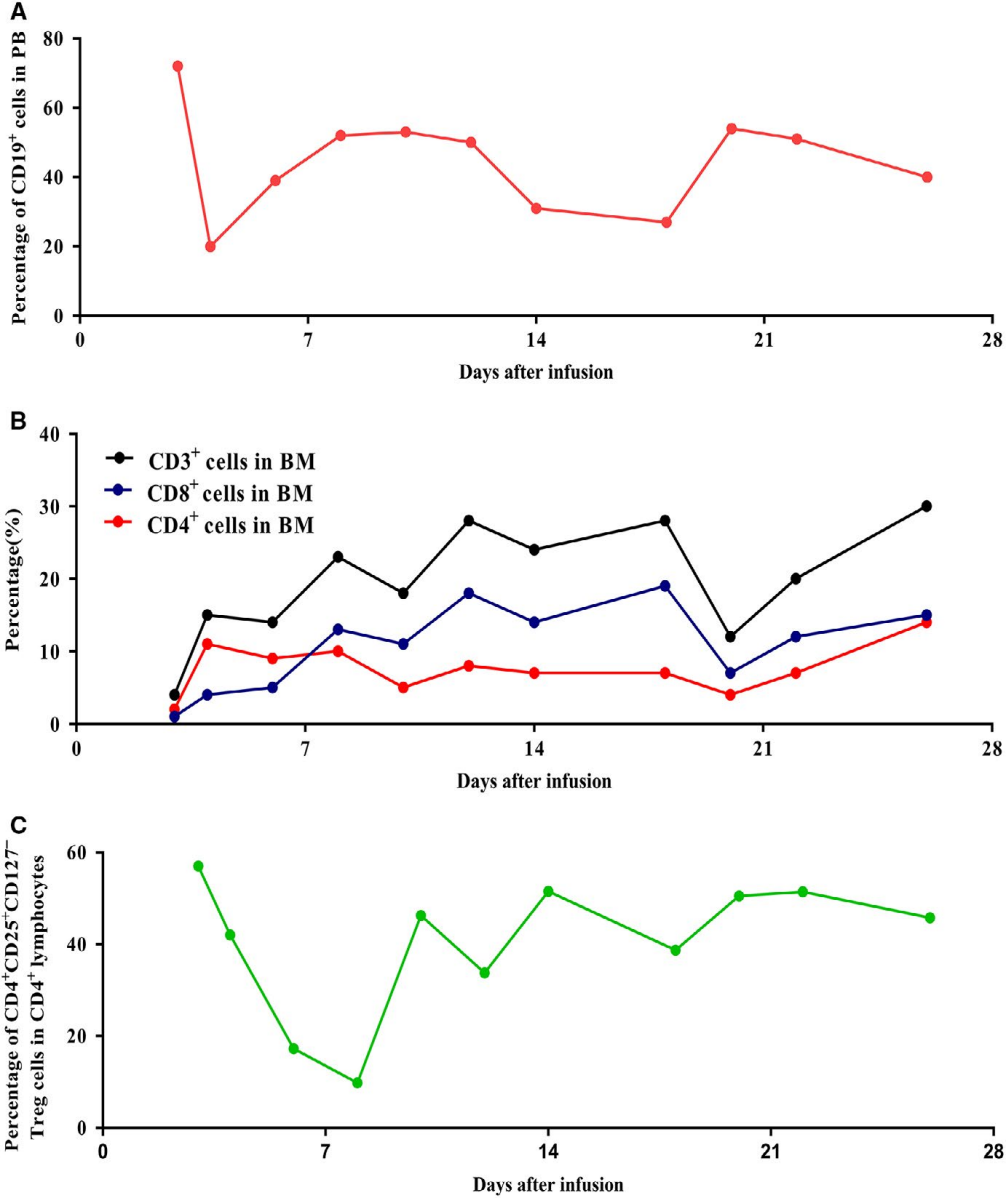
The dynamic variation of CD19 (A), CD3, CD8, and CD4 lymphocytes (B), and Treg cells (C), in peripheral blood during the CART‐19 infusion

Further, we tested the phenotype of lymphocytes in PB, we found the CD19^+^ B‐lineage cells and CD4^+^CD25^+^CD127^−^ Treg cells were down‐regulated at the early stage of infusion, rapidly raised back to a high level and sustained during the progress period, Figure 5A,C. Percentage of CD3^+^ T cells were restored, and CD8^+^ cells were up‐regulated significantly at the early stage of infusion. After, the percentage of CD4^+^ cells were increased more rapidly than CD8^+^ cells, Figure 5B.

### 3.3 Toxicities

**Figure 6:**
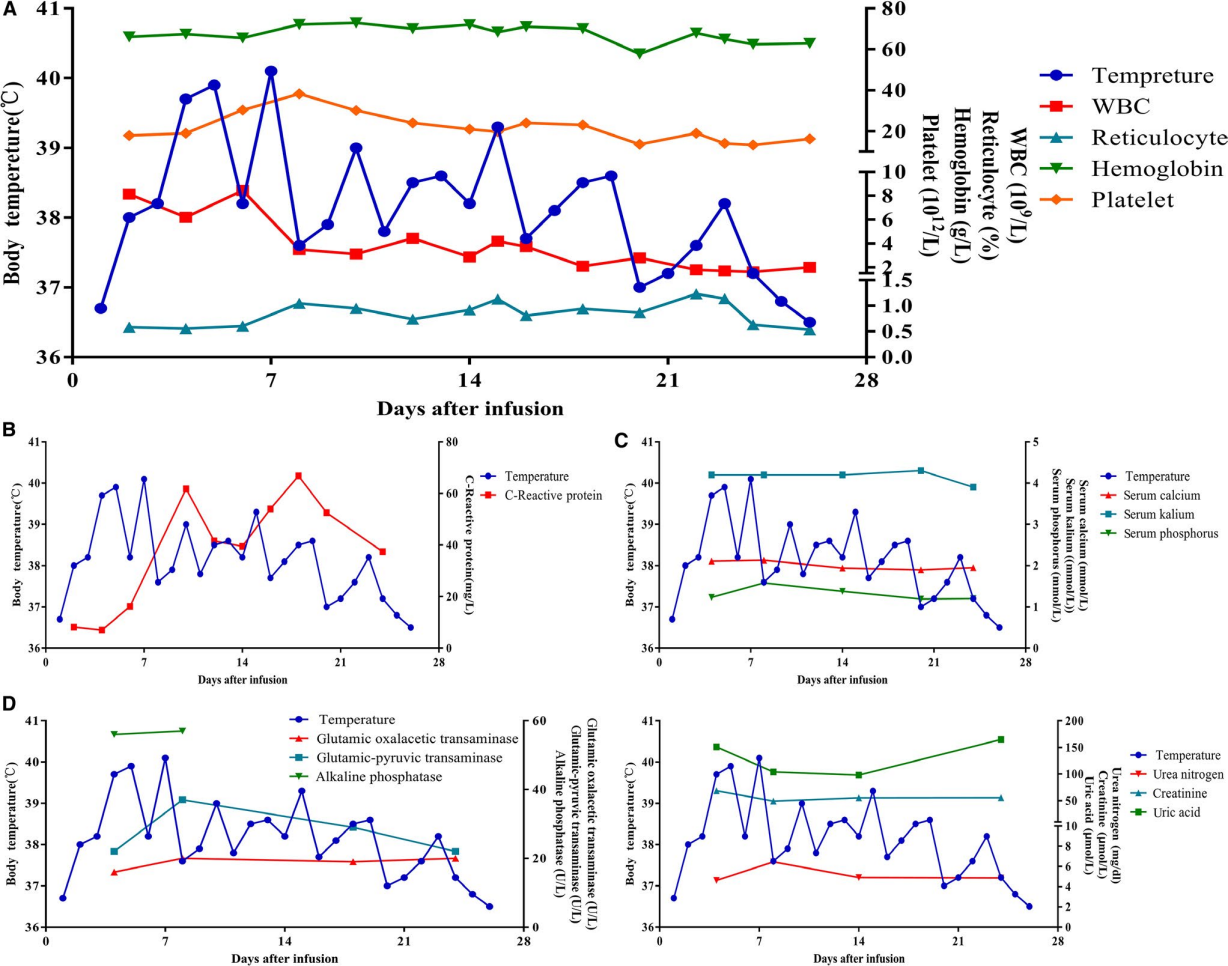
The dynamic variation of body temperature and blood cell count (A), C‐reaction protein (B), serum electrolyte (C), serum hepatorenal function (D&E) during and after the CART‐19 cell infusion

Fever occurred at the third day after cell infusion, and last 24 days with the highest temperature of 40.1°C, and relieved after symptomatic treatment, Figure 6A. The patient's pancytopenia ameliorated on 6th day. Similar as above, the percentage of CD71^+^ cells up‐regulated in CD45^−^ gate but not CD45^+^.

The serum level of C‐reaction protein raised around 10th day dramatically, has no relationship with body temperature. And signs of infection were not found during the whole period after infusion, Figure 6B. The serum level of electrolyte, hepatorenal function were observed, no significant changes have been found, Figure 6C‐E. Safely, tumor lysis syndrome did not occur in this RS patient after infusion.

**Figure 7:**
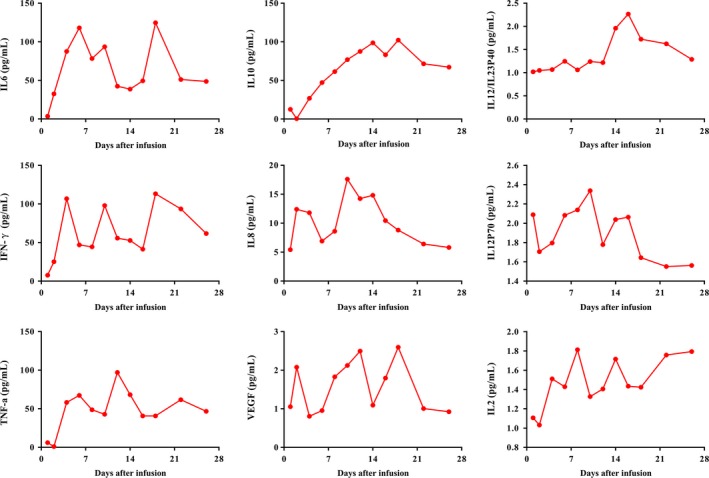
The dynamic variation of various serum levels of cytokine after CART‐19 cell infusion

To evaluate the status of cytokine release syndrome of the patient, we monitored the serum level of cytokines by Elisa. Most of the serum level of cytokines were fortified after cells infusion immediately, and maintained at a high level. Some cytokines were zigzag fluctuated and declined at the beginning of the third week. Together, the patient suffered grade I cytokine release syndrome (CRS) (Figure 7).

## 4 DISCUSSION

Approximately 15% of patients with CLL transform eventually to RS, a highly aggressive phase of CLL, which morphologically mimics diffuse large B‐cell lymphoma (DLBCL) and frequently has a dismal outcome with a median survival of 5‐8 months.^12^, ^13^ According to a multivariate analysis based on the clinical data from MD Anderson Cancer Center, the patient enrolled in our clinical trial belongs to the high‐risk group.^14^

Unfortunately, this patient enrolled in our clinic trial was primary resistant to R‐CHOP. Hematopoietic stem cell transplantation is an alternative approach for conquering RS, but can only be as post‐remission therapy. The harsh condition limits the application of HSCT in RS patient's clinical treatment. Only 10%‐15% RS patients have the chance to receive HSCT in clinic. Although some novel agents perform promising prospect of efficient activity in RS, the data should be identified in further research.^1^ Together, stagnant advance was made in the treatment of RS currently.

CART‐19 cells infusion is an alternative promising treatment in B‐cell original tumor. Recent publication shown that up to 83% relapsed B‐ALL patients achieved a CR status.^11^ However, based on previous reports,^15^ much weaker achievements were got in aggressive lymphoma of CART‐19 administration, the CR rate was less than 40% and ORR was around 70%.^15^ The efficacy of CART‐19 therapy in RS patient was unreported presently.

In current case report, the high‐risk group patients were resistant to multiple regimens after DLBCL type RS transformation, and were unfit to proceed stem cell transplant. This patient was bearing three high‐risk factors: 1, 772U/L of serum LDH; 2, count of PLT was 47.1 × 10^9^/L; 3, received non‐standard chemotherapy.

Circulating CART‐19 cells were monitored by PCR, as we do not have appropriate antibody which could bind to anti‐CD19 CAR molecule. Both PB and BM were monitored. The peak of anti‐CD19 CAR gene expression in PB was observed on 11th day after immunocytes infusion, and anti‐CD19 CAR gene expression maintained in a high level till 20th day. Interestingly, we detected a high‐level gene expression of anti‐CD19 CAR in BM on 30th day. Unfortunately, we failed to collect the following data due to the patient refuse to stay in hospital on Chinese spring festival. These data indicated that the infused CART‐19 cells could proliferate in host and capable of infiltrating into BM.

CD19‐CART cells have shown its powerful efficacy on CD19^+^ leukemia cells. This multiple chemotherapy‐resistant patient achieved a great tumor burden reduction for more than 7 weeks after CART‐19 cells infusion. Encouragingly, we found the patient achieved a hematopoietic recovery before 17th day. The population of CD71^+^ cells from CD45^‐^ gate significantly raised to 22.56% from 5.32%, and last for 7 weeks. Cells from CD45^+^ gate did not show any difference from the CD45^+^ cells before infusion. For personal reasons, the patient refused to get further immunotherapy, and survived for total 16 months since RS transformation. Nevertheless, we predicted the tumor burden of patient could be further reduced if he stayed in the trial continued to receive CART‐19 immunotherapy.

Juan et al reported a DLBCL‐type RS patient achieved CR after receiving continuous Blinatumomab monotherapy for 35 days.^16^ Theoretically, CART‐19 cells have superiority over Blinatumomab, due to more aggressive direct killing function. In Juan's case, after receiving a continuous and high dosage of Blinatumomab, the patient suffered from a tremors and encephalopathy during Blinatumomab administration.^16^ Recent data from a clinical study had proved that patients with high‐tumor load were more prone to develop severe CRS and neurotoxicity in process of CAR‐related therapy. Moreover, the patients with low‐tumor burden also had better prognosis.^11^ These results indicate that the prognosis was largely depending on the balance of activity and adverse effect. In other words, RS patients might be benefit from more aggressive strategy, high dosage of immunocytes and more frequent infusion.

Immunogenicity and immunosuppression like a teeterboard in host immunity, cancer cell targeting immunocytes enhances the immunogenicity of carcinoma cells could not reverse tumor microenvironment (TME) due to immunosuppression.^17^ In CART panel, immunosuppression would weaken the CART cells efficacy, generation of immunosuppressive TME might consequent on: 1, indiscriminate infected CAR‐expressing Treg cells; 2, immunosuppressive environment induced by cytokines secretion; 3, endogenic Treg cells. We found elevated Treg cells population in PB on the 10th day after CART infusion. Immunosuppressive Treg cells might inhibit the CART cells tumor killing function. Currently, several mechanisms were reported to reveal why Treg cell increased after T‐cell infusion. Engrafting Treg cells with chimeric antigen receptor were supposed to be a promising strategy for autoimmunity disease,^18^ nevertheless CAR‐expressing Treg cells were impeded the proliferation and responsiveness of effect T cells. In this case, CART‐19 cells were generated in both CD3^+^CD8^+^ and Treg cells; these artificial CAR‐expressing Treg cells could infiltrate and accumulate in TME. Treg‐derived cytokines may indirectly hamper the efficacy of CART‐19 on the induction of regulatory T cells,^19^, ^20^, ^21^ such as VEGF, IL‐2, IL‐10, IL‐6, IFN‐γ. In PB of the patient, the cytokines were significant increased after CART‐19 cells infusion, like IL‐2, IL‐10, IL‐12/IL‐23p40. Indoleamine 2,3‐dioxygenase (IDO) regulates host immunity associated with tryptophan metabolism.^22^ Ninomiya et al^23^ reported that IDO plays an important direct role in inhibiting CD19‐CART activity. Here, we hypothesized the immunocytes with inhibitive function infiltrated into TME were elevated paralleled with PB. So, blockage of immunosuppressive function of TME may enhance effecacy of CART‐19 cells. Certain studies support our hypothesis, anti‐PD‐L1 could augment the antitumor activity of tumor‐directed immunocytes in inhibiting tumor growth.^24^, ^25^ Schuster et al reported a refractory DLBCL patient of primary mediastinal origin with extra‐nodal involvement of small intestine. The patient's tumor cells strongly expressed PD‐L1. The patient continues to be clinically well for 12 months after CART‐19 plus anti‐PD‐L1 administration.^26^ Hence, inhibition of immunosuppressive factors could enhance the activity of CART‐19 in malignancy, especially solid tumor, thanks to more complicated TME of neoplasm have than leukemia.

As we described previously, there is a balance between activity and adverse effect. Severe adverse effects, like CRS, neurotoxic events and tumor lysis syndrome did not occur in the patient expect from a governable fever. In the following case, we will increase the dosage of CART‐19 cells and raise infusion density.

This is the first case that illustrates CART‐19 cells activity in refractory DLBCL‐type RS. Together, CART‐19 therapy is a promising strategy for DLBCL‐type RS, which also needs further clinical studies to optimize protocol of infusion to improve the outcome and reduce the toxicity. Moreover, it is reported that combined with blockage of PD‐1/PD‐L1 axis could enhance the activity of CART‐19 cells.

## CONFLICT OF INTEREST

The authors declare that they have no competing interests.
